# Archaea Dominate Ammonia Oxidizers in the Permian Water Ecosystem of Midland Basin

**DOI:** 10.1264/jsme2.ME13022

**Published:** 2013-09-04

**Authors:** Yiguo Hong, Wang Youshao, Feng Chen

**Affiliations:** 1 State Key Laboratory of Tropical Oceanography, South China Sea Institute of Oceanology, Chinese Academy of Sciences, Guangzhou, P. R. China, 510301; 2 Laboratory of Marine Ecosystem and Biogeochemistry, Second Institute of Oceanography, SOA, Hangzhou, 310012, China; 3 Institute of Marine and Environmental Technology, University of Maryland Center for Environmental Science, Baltimore, Maryland, 21202 USA

**Keywords:** Ammonia oxidizer, Permian water, Underground basin

## Abstract

We investigated the existence and characteristics of ammonia oxidizers in Permian water from Midland Basin. Molecular surveys targeting the *amoA* gene showed that only ammonia-oxidizing archaea (AOA) exist and have potential activity in this special environment. In contrast, no ammonia-oxidizing bacteria (AOB) were detected in the water. Phylogenetic analysis indicated that 72–89% of the total screened AOA clones were affiliated with those found in underground water, and 10–24% of the AOA clones were related to those found in marine water or sediments. Our results indicate AOA might be the most abundant ammonia-oxidizing microbes in this ecological niche.

The conversion of ammonia to nitrite is an important microbiological process for nitrification, which is performed by the key ammonia monooxygenase (AMO) enzyme encoded by the *amo* gene (23). It has traditionally been assumed that this step is carried out mainly by autotrophic ammonia-oxidizing bacteria (AOB) of the β- and γ-subgroups of proteobacteria (16, 21, 22). However, this view has recently been changed by the discovery of the *amoA* gene in archaea populations, thus raising the prospect of the presence of ammonia-oxidizing archaea (AOA) in different ecosystems (10, 17, 21, 29, 32). In fact, AOA were found to be more abundant than AOB in a range of terrestrial and marine ecosystems, suggesting that AOA can play a significant role in nitrogen biogeochemical cycling (2, 4, 10, 21, 32). There is increasing evidence that environmental conditions, such as pH (20), salinity (19, 24), and especially ammonium availability (7, 15, 18), can affect the distribution, abundance and activity of AOB and AOA. Although AOA and AOB have been investigated in diverse environments, their distribution and diversity in Permian groundwater remain unexplored.

The Permian Basin is a unique ecosystem which contains the remnant of an ancient ocean that existed during the Permian time (~250 million years ago) (33). The Permian Basin is a sedimentary basin largely contained in the western part of the U.S. It reaches from just south of Lubbock, Texas, to south of Midland and Odessa, extending westward into the southeastern part of the adjacent state of New Mexico. It is so named because it has one of the world’s thickest deposits of rocks from the Permian geologic period. In the long historical period, the Permian basin received outside water gradually through the penetration of surface water or input of deepwater (1). A significant feature of the sample is high nitrate concentration and relatively low ammonia concentration (Table S1), which draw attention to the importance of nitrification. It could be presumed that ammonia oxidizers play important roles in the transformation from ammonia to nitrate. The goal of this study was therefore to investigate the existence, abundance and activity of ammonia oxidizers in Permian water based on the *amoA* gene and to evaluate their potential function in nitrogen transformation in this specific underground water.

For the above purposes, Permian water samples were collected from a location in the Pecos Cenozoic Trough in Imperial, Texas (latitude 31, 16′, 16.93″ N; longitude 102, 40′, 48.35″W) (Fig. S1) in December 2010 and July 2011, respectively. After sampling, the water was fixed with HgCl_2_ immediately for hydrochemical analysis. For preparing samples for DNA analysis, 1L Permian water was filtered through a 0.22 μm pore-size membrane filter and the folded filtered membrane was placed into a 2 mL tube. The same procedure as above was repeated to prepare samples for RNA analysis. A difference was that the folded filtered membrane was placed into a 2 mL tube containing RNA later solution (Life Technologies, Carlsbad, CA, USA). All membrane samples were kept at −80°C.

As displayed in Table S1, the salinities of the two Permian water samples were 17.5‰ and 15.5‰, respectively, equivalent to approximately half of the average salinity of the ocean. The water contained a high concentration of bicarbonate, which can be supplied as a carbon source for autotrophic microbes. The average concentration of ammonia in Permian water is 0.19 μM, lower than that in the marine euphotic zone (0.3 μM), but higher than that in the marine aphotic zone (0.01 μM) greatly. The nitrite concentration in Permian water (average 0.77 μM) exceeds that in seawater (5, 14). The aerobic environment may contribute to the accumulation of nitrate, because aerobic conditions do not favor the processes of denitrification and anammox. The mean ratio of N/P is 20.95, with no major deviation from general terrestrial or marine ecological environments. These hydrological parameters offer an ambient background for analyzing the interactional relationship between ammonia oxidizers and environmental conditions.

For performing the molecular investigation, DNA and RNA were extracted using a PowerWater DNA Isolation Kit and PowerWater RNA Isolation Kit (MOBIO Laboratories, Carlsbad, CA, USA) with the standard protocol. Three specific primer sets were used for *amoA* gene amplification and qualification: Arch-amoAF and Arch-amoAR (10) for AOA, amoA-1F and amoA-2R (23) for β-AOB and A189-for/A682-rev (22) for γ-AOB. In addition, the bacteria and archaea were detected with general primer sets 27f/1492r (9) and 21f/852r (8) respectively. All PCR products of the *amoA* gene were purified by cutting gel bands with the Qiagen II Gel Extraction Kit (Qiagen, Hilden, Germany) and then cloned into pCR 2.1 TOPO T-vector (Invitrogen/Life Technologies, Carlsbad, CA, USA) to construct the gene libraries. The positive clones TOPO-1 and TOPO-2 with an inserted *amo*A gene fragment were selected as standards for real-time quantitative PCR (q-PCR) to detect the *amoA* gene abundance of AOA and AOB, respectively. Q-PCR was run on an Eco Real-Time PCR System (Illumina, San Diego, CA, USA) using power SYBR Green PCR Master Mix (Applied Biosystems/Life Technologies, Carlsbad, CA, USA), according to the manufacturer’s instructions. Standard curves were prepared from the serial dilution (10^8^–10^2^ *amoA* copies by decimal dilution series) of plasmids containing environmental archaeal and beta-proteobacterial *amoA* gene sequences. The PCR efficiencies were 87–94% (average 90%) for archaeal *amoA* and 92–96% (average 93%) for beta-proteobacterial *amoA*. Correlation coefficients (R^2^) for both assays averaged 0.98 (standard deviation of 0.01).

Fig. S2 shows that the amplification of AOA was positive, but the amplifications of β- and γ-AOB were both negative, even under low stringency PCR conditions. Furthermore, q-PCR detection showed that the abundance of AOA was approximately 3.4×10^4^ copies mL^−1^ water, but the abundance of AOB was not detectable (the detection limit of q-PCR was approximately 10 copies mL^−1^ in positive control experiments for both AOA and AOB) (Table 1). The finding that only AOA, not AOB, were detected in Permian water with both general PCR amplification and real-time q-PCR detection suggests that AOA are the dominant and even exclusive contributors to ammonia oxidation in Permian underground water.

To gain insight into the diversity of AOA in Permian water, we constructed two clone libraries based on *amoA* amplifications from the two Permian water samples. A total of 91 clones from these two clone libraries were screened and sequenced. BLAST results revealed that all 91 clones were closely affiliated with known AOA. With molecular analysis tools (6, 26, 27), further phylogenic analysis exhibited that the 91 clones were grouped into three clusters: fresh water, marine and soil (Fig. 1). The majority (80%) of sequences retrieved from Permian water fell into the freshwater cluster and 20% sequences were in the marine cluster, but only one sequence belonged to the soil cluster. The Permian water sequences in the freshwater cluster were closely related to the clones from underground or river water; for example, the clones from the lower Mississippi River (GQ906666), low-nutrient groundwater (FJ543284), Dongjiang River (JQ312231), and Idaho Falls underground water (FJ543359). The sequences contained in the marine cluster were most similar to clones found in the Black Sea (EF414231), the Northeast Japan Sea (AB289385), deep sea (EU885581) or estuarine sediments (EU025152), and the deep sea hydrothermal vent (EU427963). Using a 3% cutoff at the DNA sequence variation to define an OTU, the non-asymptotic rarefaction curves showed that the OTU number would be higher if more clones were sequenced (Fig. S3).

To determine whether *amoA* genes from AOA and AOB communities are actively transcribed in Permian water and whether transcriptions are correlated with gene abundance, we extracted total RNA from two samples and constructed two *amoA* cDNA libraries. Then, 20 *amoA* cDNA clones were randomly selected and sequenced. The phylogenic relationships of the deduced amino acid sequences were close and were all clustered within AOA freshwater cluster (Fig. S4), suggesting that the freshwater cluster AOA possibly play an active role in ammonia oxidation in the Permian water. Using a 3% cutoff at the DNA sequence variation to define an OTU, the number of OTUs of cDNA clones has reached the maximum. With the reverse transcription q-PCR method, we detected that the transcript abundance of AOA was approximately 2.3×10^3^ copies mL^−1^ (Table 1), while the transcripts of AOB were under the detection limit. Corresponding to the transcriptional activity of AOA communities, the ammonia oxidation rate was detectable in Permian water. A set of experiments with NaClO_3_ as an inhibitor were performed to determine the ammonia oxidation rate. Because NaClO_3_ can inhibit the oxidation reaction from nitrite to nitrate, nitrite could be accumulated when NaClO_3_ was supplied to the reaction system (13). Thus, the increase of nitrite can be regarded as the rate of ammonia oxidation. Fig. 2 shows that the ammonia oxidation rate was approximately 0.337 μM d^−1^. However, ammonia oxidation activity was not completely inhibited by ammonia oxidation inhibitor allylthiourea (ATU) (Fig. S5). A previous study also reported that the nitrification rate in the California Current dominated by AOA was inhibited only partially by ATU (25) at the level known to completely inhibit cultivated AOB (11, 28, 24). The different response of AOB and AOA to ATU is possible from their different mechanism of ammonia oxidation (28, 31). The difference in the relative abundance and expression of bacterial and archaeal ammonia oxidizers may be due to the different ability to adapt to the niche. Previous studies (12, 30) have demonstrated that ammonia concentration influences their abundance and activity and AOA prefer an environment with a low ammonia concentration. Martens-Habbena *et al.* (18) showed that *Nitrosopumilus maritimus* SCM1 have a high affinity for ammonium, as much as 200-fold higher than that of AOB, and a low substrate threshold of 10 nM NH_4_ or less. The available ammonia in Permian water was very low (mean 0.2 μM). The source of the ammonia may be leakage of surface water, or organic minerals contained in the surface water (1). Based on the finding of AOA’s markedly high specific affinity for ammonia (16), the low level of available ammonia in Permian water is maybe an important factor contributing to the survival of AOA rather than AOB. In addition, experimental results of PCR amplifications showed that bacteria and Achaea are both present in Permian seawater (Fig. 1), suggesting that the environment in Permian water was not favorable for the survival of Achaea or bacteria. So the difference in the abundance of AOA and AOB in Permian water possibly resulted from their own physiological and metabolic characteristics adapting to the low ammonia environment.

Interestingly, the *amoA* gene phylogenies from Permian water reveal that these sequences represent two distinct clusters (Fig. 1). Approximately 80% AOA clones in Permian water belong to the freshwater cluster, and they are closely related to the AOA sequences retrieved from underground water. Less than 20% clones are affiliated with the marine cluster. The co-existence of freshwater and marine AOA is consistent with the environments from which they were drawn and strengthen the idea that environmental niches within the AOA are reflected in the *amoA* phylogeny. It is possible that that salinity is an important factor for AOA adapting to ecological niches. Recent reports (2, 19, 24) support the idea that salinity affects the community composition of AOA.

In this study, the measured salinity in the Permian water from Midland basin is approximately half of average salinity in the ocean. The salinity of Permian water is much lower than that of the originally enwrapped sea water because evidence suggests that ancient marine salinity was similar to or higher than that of the modern sea (3). The decreased salinity is the result of mixing with fresh water penetrating from the surface continuously (1). Along with the penetrated water, microorganisms including AOA possibly entered the Permian basin. If marine cluster AOA are regarded as indigenous to the original Permian water, freshwater cluster AOA should be exogenous. Previous investigations suggested that AOA have pronounced ecological niche separation based on *amoA* gene phylogenies and the community composition of AOA in the marine environment is distinct with that in freshwater (2, 10, 25). With the change of the environment in Permian sea water, the composition of ammonia oxidizers may have changed to adapt to the new environment. Gradually, freshwater cluster AOA have taken the dominant position. However, the relationship between the molecular information and the geological features needs further study.

In conclusion, the molecular information provided strong evidence that AOA rather than AOB are responsible for the ammonia oxidation process in Permian water. Environmental conditions, such as the available ammonia concentration and salinity, possibly shaped the population structure and function of ammonia oxidizers. To our knowledge, this is the first study indicating the potential role of AOA in the environment of Permian water. Future work will address the activity of the community and examine the factors controlling ammonia oxidizers’ diversity and abundance and how to adapt to a confined environment.

The GenBank accession numbers for the *amoA* gene sequences reported here are JX311735–JX311856.

## Supplementary Material



## Figures and Tables

**Fig. 1:**
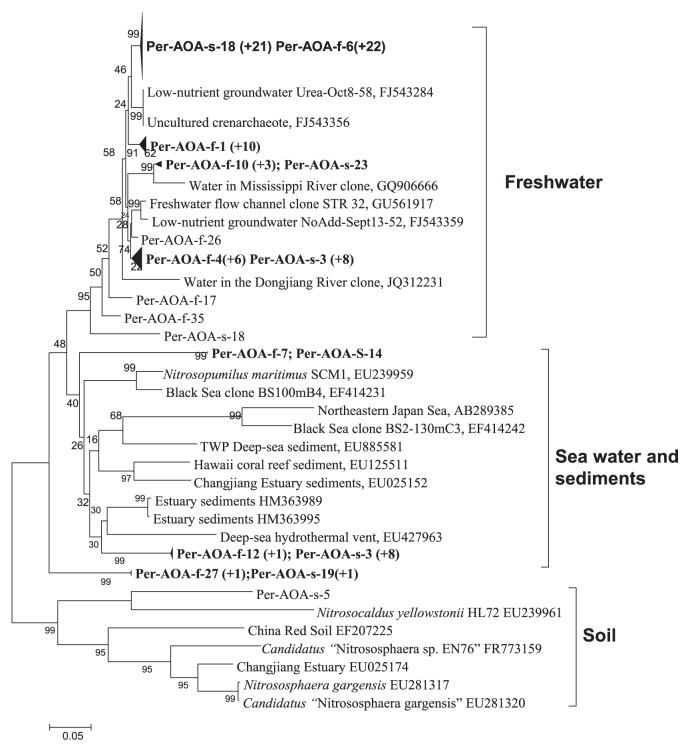
Phylogenetic relationships among archaeal *amoA* sequences from Permian underground water and previously reported environmental sequences. This tree was constructed with the neighbor-joining method based on Jukes-Cantor-corrected DNA distances and midpoint rooted. Accession numbers corresponding to the 91 sequences represented in this tree are described in Materials and methods. Scale bar represents 5% estimated sequence divergence. The relative percentage of different phylogenetic lineages. f represents the December 2010 sample and s represents the July 2011 sample. For the larger figure, see also supplementary online material.

**Fig. 2:**
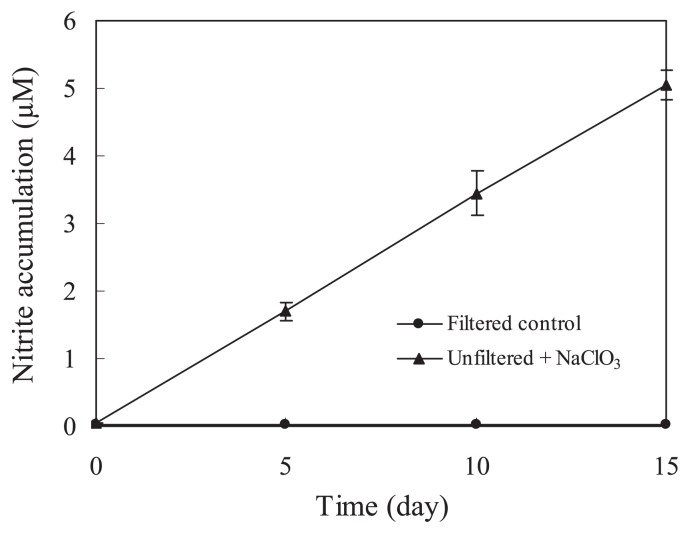
Ammonia oxidation rates by the Permian water measured by adding NH_4_^+^ (final concentration 10 μM). Inhibited bottles were spiked with the nitrite-oxidation inhibitor NaClO_3_ to achieve a final concentration of 10 mg L^−1^. Water was filtered through a 0.22 μm pore-size membrane as a control.

**Table 1:** A rchaeal *amoA* gene and β-proteobacterial *amoA* gene copy numbers in the samples from Permian water

*Sample No.*	Archaeal *amoA* gene copies mL^−1^ (s.d.)	β-proteobacterial *amoA* gene copies mL^−1^ (s.d.)	Archaeal *amoA* mRNA copies mL^−1^ (s.d.)	β-proteobacterial *amoA* mRNA copies mL^−1^ (s.d.)
Sample 1	1.96×10^4^ (7.48×10^3^)	U	1.93×10^3^ (0.82×10^2^)	U
Sample 2	4.83×10^4^ (1.39×10^3^)	U	2.68×10^3^ (3.99×10^2^)	U

U: Undetectable.
