# Supplementary material for: Archaea Dominate Ammonia Oxidizers in the Permian Water Ecosystem of Midland Basin
Source: Microbes Environ. 2013 Sep 4;28(3):396–9. doi: 10.1264/jsme2.ME13022 (PMC4070961; doi:10.1264/jsme2.ME13022)
Supplement: Supplementary file 1 [file 28_396_s1.pdf]

Supplementary materials

Archaea dominate ammonia-oxidizer in the Permian water ecosystem of Midland Basin

Yiguo Hong[1,2], Youshao Wang[1], Feng Chen[3]

[1]State Key Laboratory of Tropical Oceanography, South China Sea Institute of Oceanology, Chinese Academy of Sciences, Guangzhou, P. R. China, 510301; [2]Laboratory of Marine Ecosystem and Biogeochemistry, SOA, Second Institute of Oceanography, SOA, Hangzhou, 310012, China; [3]Institute of Marine and Environmental Technology,University of Maryland Center for Environmental Science, Baltimore, Maryland, 21202 USA.

1. Detail materials and methods:

DNA, RNA Extraction and PCR Amplification- DNA and RNA were extracted using PowerWater® DNA Isolation Kit and PowerWater® RNA Isolation Kit (MOBIO Laboratories, USA) with standard protocol. Three pairs of primers were employed for amoA gene amplification and qualification: Arch-amoAF and Arch-amoAR for AOA *amoA*, amoA-1F and amoA-2R for β-AOB *amoA*, and A189-for/A682-rev for γ- AOB *amoA*. In addition, the bacteria and archaea were detected with general primer pair 27f/1492r and 21f/852r respectively. The optimized PCR reaction mixture in a final volume of 50 µl contained the followings: 1 µl DNA (30~50 ng μl^{-1}), 0.5 µl bovine serum albumin (100 mg ml^{-1}, Roche), 5 µl 10×PCR Buffer and 1.5 µl $MgCl_2$ (50 mM, Promega), 1 µl of dNTPs (10 mM, Invitrogen), 1 µl of forward and reverse primers (20 µM), and 0.25 µl of Taq polymerase (Platinum Taq DNA Polymerase kit, invitrogen). PCR programs were as followings: phase I: 95°C for 3 min; 15 cycles of 95°C for 45 s, 48°C for 1 min, followed by 72°C for 1 min; phase II: 95°C for 3 min; 28 cycles of 95°C for 45 s, 53°C (for AOA *amoA*) or 55°C (for β-AOB *amoA*) or 53°C γ-AOB *amoA*) for 1 min, followed by 72°C for 1 min; and finally 72°C for 10 min. Platinum® Taq DNA Polymerase is a convenient and reliable "hot start" thermostable DNA polymerase, providing increased sensitivity, specificity, and yield in PCR. So it can amplify the DNA in very low concentration in the reaction system.

Cloning, Sequencing and Phylogenetic Analysis- All PCR products of the *amoA* gene were purified through cutting gel bands with the Qiagen II Gel Extraction Kit (Qiagen, Hilden, Germany) and then cloned into the 2.1 TOPO T-vector (Invitrogen) for constructing the gene libraries. The size of cloned DNA fragment was determined by PCR amplification with the

primer set M13F(TGTAAAACGACGGCCAGT) and M13R(CAGGAAACAGCTATGACC). Sequencing was performed with the BigDye Terminator Kit (Applied Biosystems, Foster City, CA) and an ABI Prism 3730 DNA analyzer. The DNA sequences were examined and edited by MEGA 4.0 software and then checked for chimera formation using the Check Chimera program of Ribosomal Database Project or manually. For the 16S rRNA gene, DNA sequences were manually compiled and aligned using the ClustalW. Phylogenetic trees were constructed by MEGA 4.0 with the neighbor-joining and maximum parsimony methods. Bootstrap resampling analysis on 500 replicates was performed to estimate the confidence of the tree nodes.

Quantitative PCR Assay-The *amoA* gene abundance was determined by real time quantitative PCR (*q*-PCR) on an Eco Real-Time PCR System (Illumina, Inc. San Diego, USA) using power SYBR Green PCR Master Mix (ABi, USA), according to the manufacturer's instructions. Standard curves were prepared from a serial dilution (10^8~10^2 *amoA* copies by decimal dilution series) of plasmids containing environmental archaeal and beta-proteobacterial *amoA* gene sequences (clone TOPO-1 for AOA and clone TOPO-2 for AOB). The PCR efficiencies were 87~94% (average 90%) for archaeal *amo*A and 92~96% (average 93%) for β-proteobacterial *amoA*. Correlation coefficients (R^2) for both assays averaged 0.98 (standard deviation of 0.01).

Ammonia oxidation rate measurements-Ammonia oxidation rate measurements were performed for the sample. Four 500 ml bottle incubations were conducted: two experimental bottles, one inhibited bottle and one filtered control bottle. Inhibited bottles were spiked with the nitrite-oxidation inhibitor $NaClO_3$ to achieve a final concentration of 10 mg l^{-1}. Water for filtered controls was filtered through a 0.22 μm pore-size membrane. Each bottle was spiked with 0.5 ml NH_4^+ (10 mM). Bottles were incubated in a darkened in incubator, and replicate 50 ml samples were removed from each bottle at time points of 0, 12, 24 and 36 h, immediately frozen, and stored at -20 °C until analysis. Samples were later analyzed for nitrite concentration. The ammonia oxidation rate can be calculated from increase of nitrite. Nitrite was assayed relying on a diazotization reaction originally described by Griess (3).

In addition, the inhibitory experiment was performed with ammonia oxidation inhibitor allylthiourea (ATU) in a final concentration of 10 mg l^{-1}. Ammonia was determined with idophenol blue spectrophotometric method as described elsewhere (1).

Table S1. Chemical and physical properties of Permian seawater from Midland Basin.

Chemical or physical properties	Sample 1	Sample 2	Seawater Mean*
Salinity (‰)	17.5	15.5	34
pH	7.00	7.0	7.9~8.2
Bicarbonate as $CaCO_3$ (mg/L)	268	235	
Calcium as Ca (mg/L)	860	920	410
Magnesium as Mg (mg/L)	231	238	1,310
Sodium and Potassium (mg/L)	2179	2355	10,900, 390
Sulfate as SO_4 (mg/L)	2509	2505	2740
Chloride as Cl (mg/L)	3551	3208	19,700
Iron as Fe (mg/L)	0.22	0.20	<0.02
Hydrogen Sulfide (mg/L)	0.0	0.0	
Nitrate (μmol/L)	258	291	7 euphotic zone<br>31 aphotic zone
Ammonia (μmol/L)	0.18	0.21	0.3 euphotic zone<br>0.01 aphotic zone
Nitrite as N (μmol/L)	0.62	0.91	0.1 euphotic zone<br>0.006 aphotic zone
Phosphate as (μmol/L)	12.3	14.2	1.0
N/P ratio	21.4	20.5	15-16

*Referenced from (2) and (4).

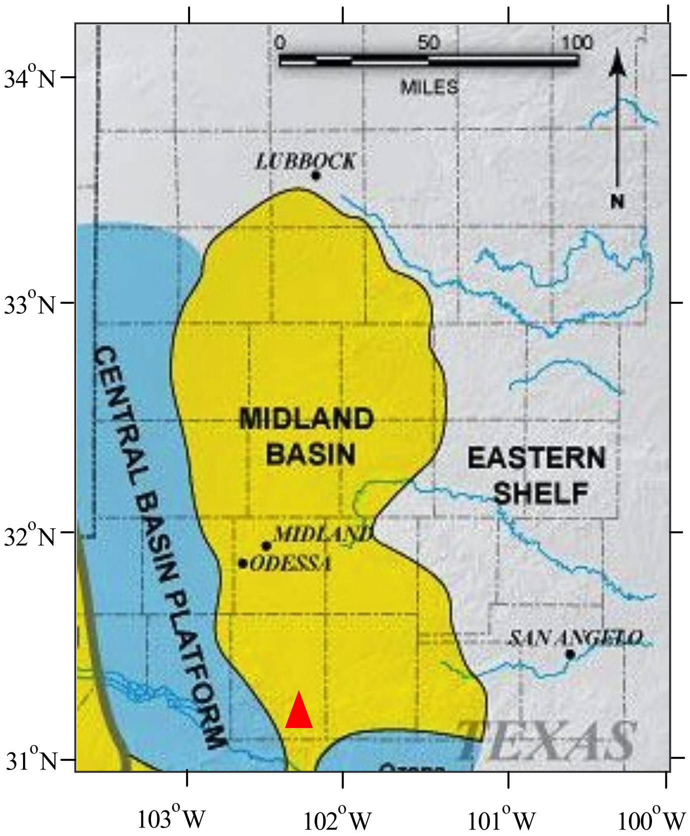

Fig. S1 The map of Midland Basin and the sampling location (red triangle)

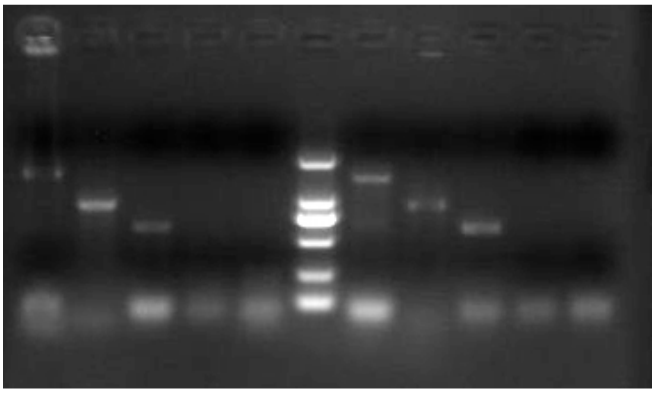

Fig. S2 PCR detection of AOA and AOB in Permian undergroudwater. A, B, C, D and E are the PCR results for the sample collected in December 2010; F, G, H, I and J are the PCR results for the sample collected in July 2011. A and F are bacterial detection with 27f/1492r primers; B and G are archaeal detection with 21f/852r primers; C and H are AOA detection with Arch-amoAF/ Arch-amoAR primers; D and I are β-AOB detection with amoA-1F/ amoA-2R primers; E and J are γ-AOB detection with A189-for/A682-rev primers. M is the DL2000 molecular marker, which contains bands of 2, 1.5, 0.75. 0.5 0.2 and 0.1 kb from up to down, respectively).

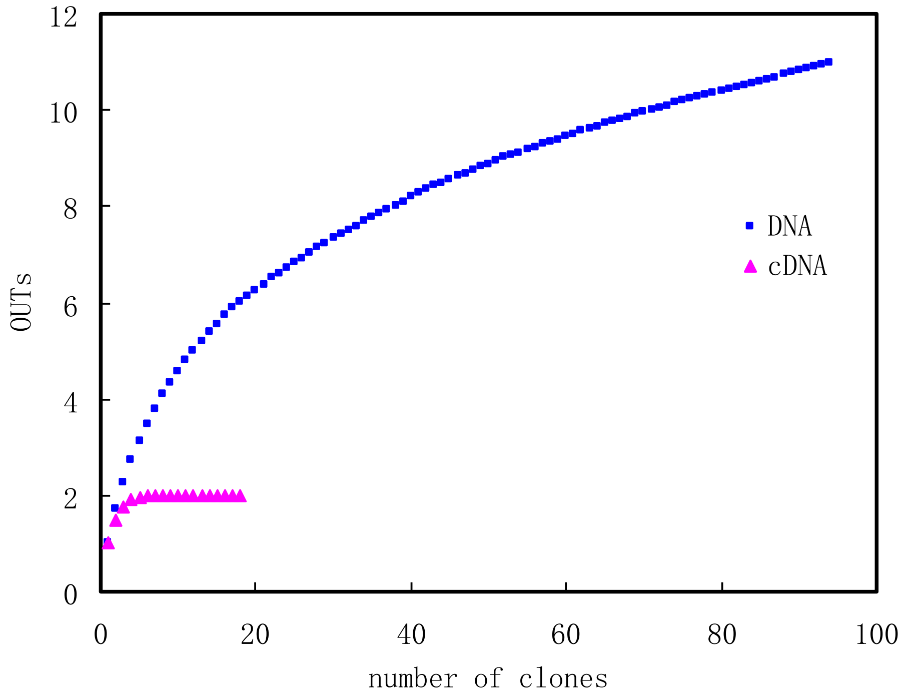

Fig. S3. Rarefaction analysis of AOA communities in DNA level and mRNA level based on *amoA* gene. DOTUR program was used with 3% nucleotide or protein sequence variation as OTU determination.

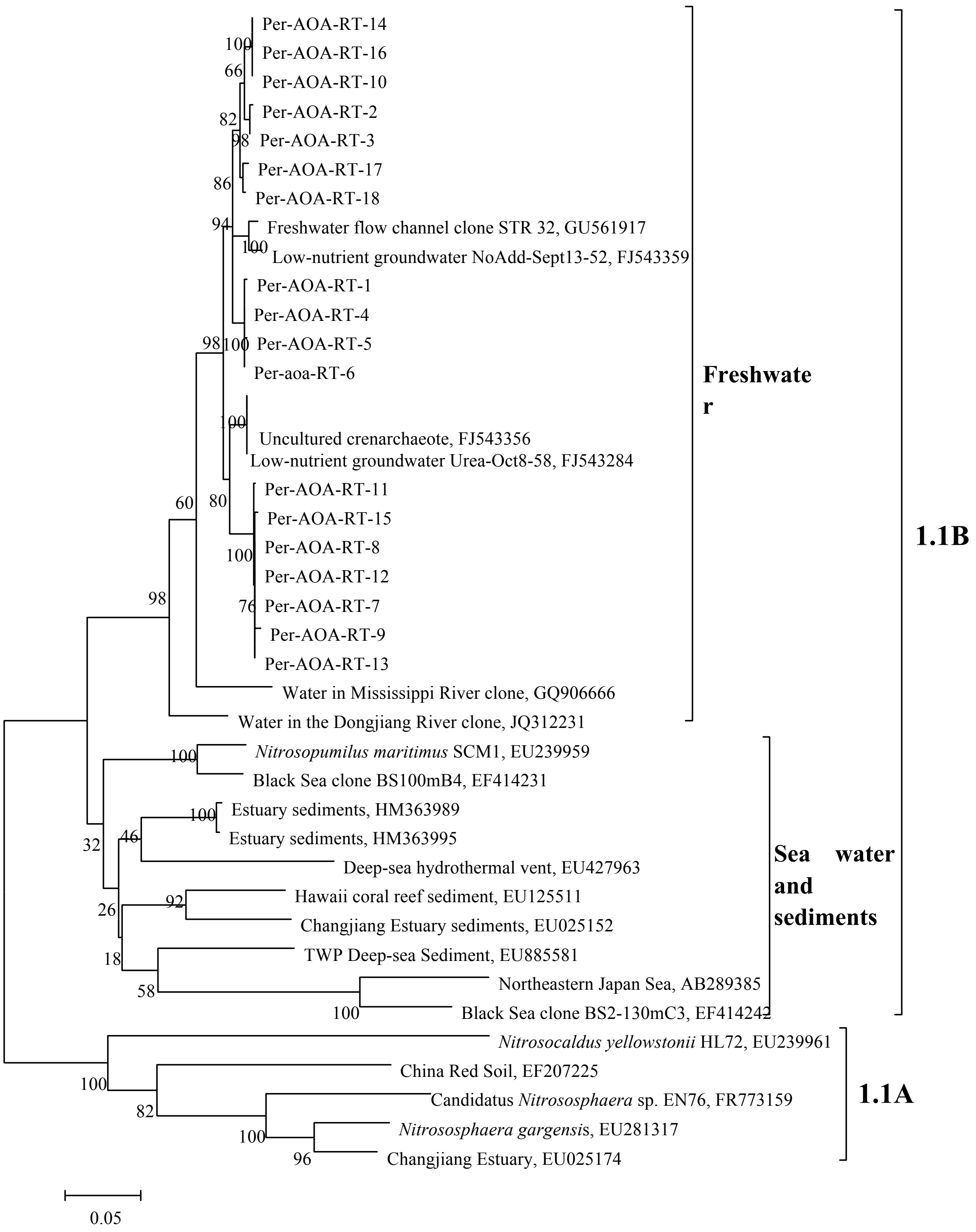

Fig. S4. Neighbor-jointing tree of the archaeal amoA sequences recovered from the RNA of Permian water. Accession numbers corresponding to the 20 sequences represented in this tree are listed in the material and methods. The scale bar is at the lower left.

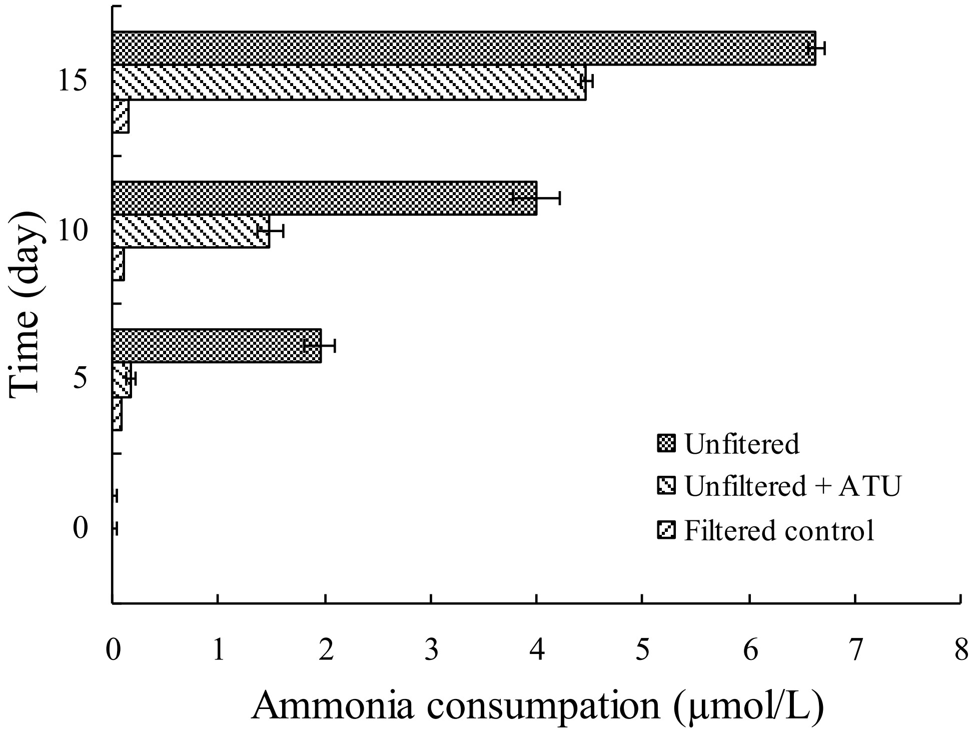

Fig. S5 Ammonia oxidation rates by the Permian water measured with allylthiourea (ATU) as inhibitor.

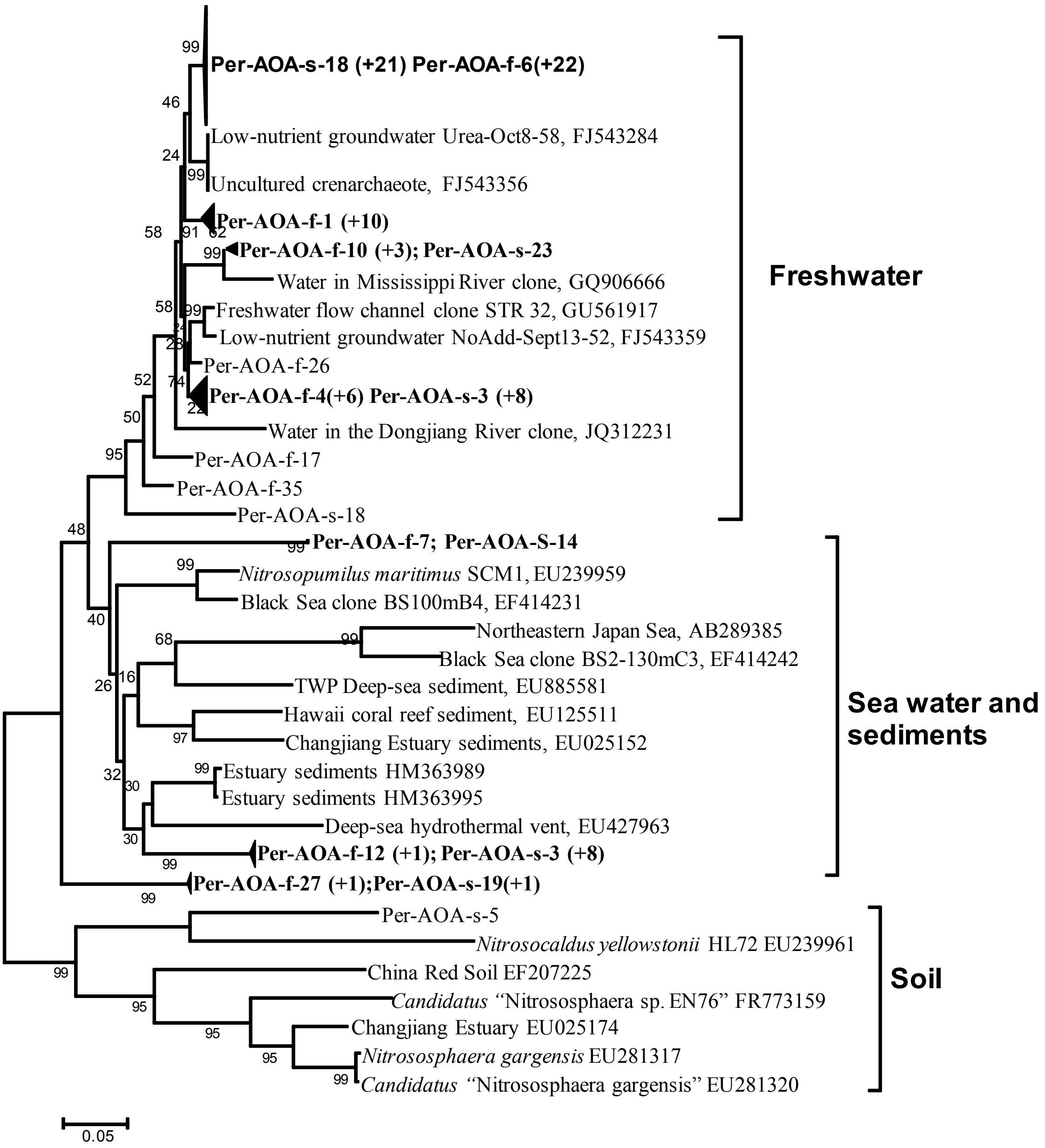

Fig. 1 Phylogenetic relationships among archaeal *amoA* sequences from Permian underground water and previously reported environmental sequences. This tree was constructed with the neighbor- joining method based on Jukes-Cantor-corrected DNA distances and midpoint rooted. Accession numbers corresponding to the 91 sequences represented in this tree are described in Materials and methods. Scale bar represents 5% estimated sequence divergence. This figure is as same as the Fig. 1 in the article.

References

1. Aminot A., Kirkwood, D.S., and Kerouel, R. (1997) Determination of ammonia in seawater by the indophenol-blue method: evaluation of the ICES NUTS I/C 5 questionnaire. Mar Chem 56: 59–75.
2. Chester, R. 2000. Marine Geochemistry, 2nd Edition, Blackwell Science, Oxford.
3. Griess, P. (1879) Bemerkungen zu der abhandlung der H.H.Weselsky und Benedikt "Ueber einige azoverbindungen." Chem. Ber. 12, 426.
4. Gruber, N. 2008. The marine nitrogen cycle: Overview and challenges, p. 1-50. *In*: D.G. Capone, D.A. Bronk, M.R. Mulholland, E.J Carpenter(ed), Nitrogen in the marine environment. Elsevier, Amsterdam
